# Subtle Presentation of Type B Esophageal Atresia

**DOI:** 10.7759/cureus.107048

**Published:** 2026-04-14

**Authors:** Susana Fortich, Keyan Mobli, Esther Ewing, Ravi Radhakrishnan

**Affiliations:** 1 Department of Surgery, University of Texas Medical Branch, Galveston, USA; 2 Department of Pediatric Surgery, University of Texas Medical Branch, Galveston, USA

**Keywords:** bronchoscopy, collis gastroplasty, esophageal atresia, gastrostomy, neonatal surgery, type b tracheoesophageal fistula

## Abstract

Esophageal atresia (EA) is a rare congenital anomaly of the foregut. Several anatomic variants have been described, ranging from isolated esophageal discontinuity to forms associated with tracheoesophageal fistula, with some variants occurring far less frequently than others. A premature infant (33 weeks, 1.5 kg) presented with a gasless abdomen and a nasogastric tube coiled at T4. Bronchoscopy revealed a Grade 1 laryngeal cleft without fistula, consistent with type A EA, and a gastrostomy was placed. The initial six-vertebral gap shortened to 2.5 over six weeks. Collis gastroplasty and thoracic anastomosis were performed. Postoperative esophagram revealed a tract to the airway, suggesting a type B EA. Repeat bronchoscopy confirmed the fistula, which was closed via a cervical approach. Recovery and follow-up imaging were uneventful. Type B EA is rare and easily missed. Repeat imaging and bronchoscopy are crucial for accurate diagnosis and management.

## Introduction

Esophageal atresia (EA) is a congenital interruption of the esophagus, most associated with a tracheoesophageal fistula (TEF). The Gross classification system describes five main types: type A (pure EA, no TEF), type B (EA with proximal TEF), type C (EA with distal TEF, most common), type D (EA with both proximal and distal TEF), and type E (H-type TEF without atresia). Type A accounts for approximately 7-8%, and type B for about 2-5% of cases, making them relatively rare compared to type C, which constitutes about 85-89% of cases [1-5].

Diagnosis is typically established by failure to pass a nasogastric (NG) tube and confirmed with radiographic imaging. Bronchoscopy is the gold standard to identify fistulae, especially proximal ones, which can be subtle and easily missed, leading to misdiagnosis, most often as type A when a proximal fistula (type B) is present [5,6]. The bronchoscopy should be performed prior to surgical repair. Proximal TEFs are often small, located just distal to the vocal cords, and may not be apparent on initial bronchoscopy or imaging, necessitating a high index of suspicion and sometimes repeat or intraoperative assessment [5,6].

Early and accurate identification of the specific EA/TEF type is crucial for optimal surgical planning and minimizing complications. We report a case of a preterm infant initially diagnosed with type A EA, which was later found to involve type B EA, demonstrating the importance of maintaining a high index of suspicion for rarer EA variants.

## Case presentation

**Figure 1:**
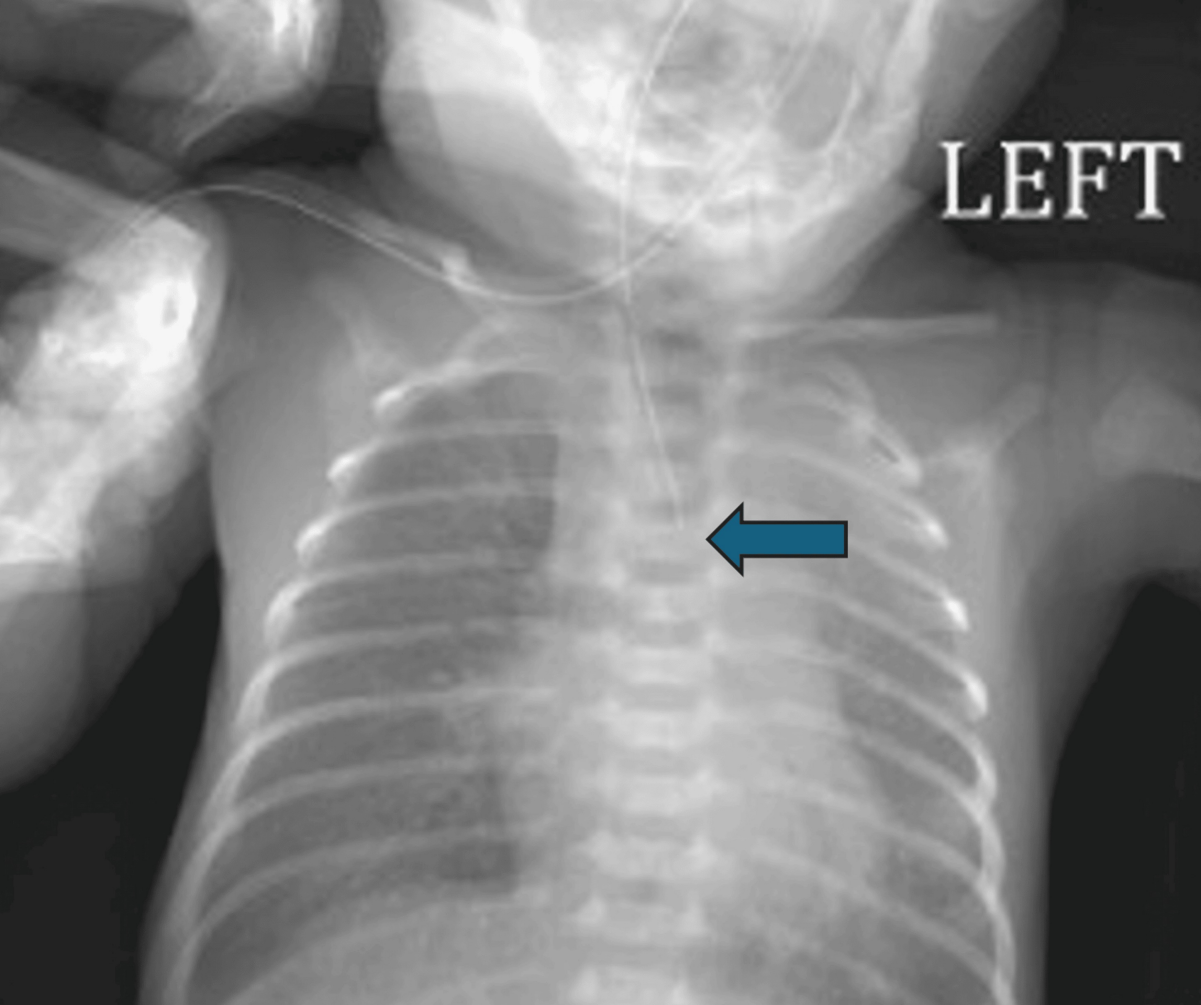
Chest x-ray demonstrating NG tube coiled at the level of T4 concerning for esophageal atresia without fistula. NG: nasogastric

A 10-month-old Hispanic male, born at 33 weeks of gestation, presented with EA and associated anomalies, including atrial septal defect (ASD), ventricular septal defect (VSD), patent ductus arteriosus (PDA), and pulmonary hypertension. The infant required immediate resuscitation at birth and was later diagnosed with EA, confirmed by a chest X-ray showing an NG tube in the upper thoracic esophagus (T4) (Figure 1).

**Figure 2:**
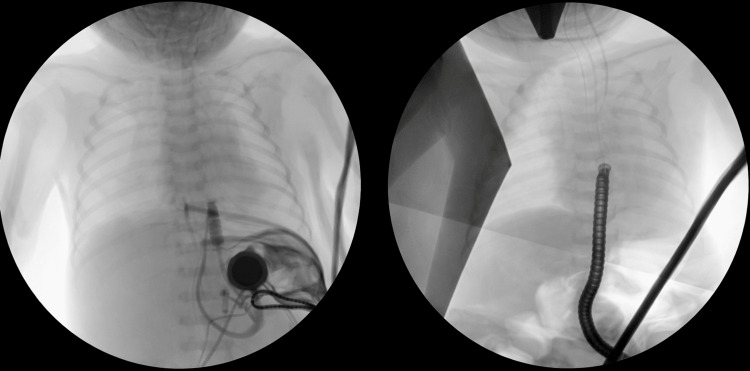
Left: The gap between the proximal and distal pouch was measured at six vertebral bodies on fluoroscopy. Right: Over the period of six weeks, the gap decreased to 2.5 vertebral bodies. ENT sees a type 1 cleft.

**Figure 3:**
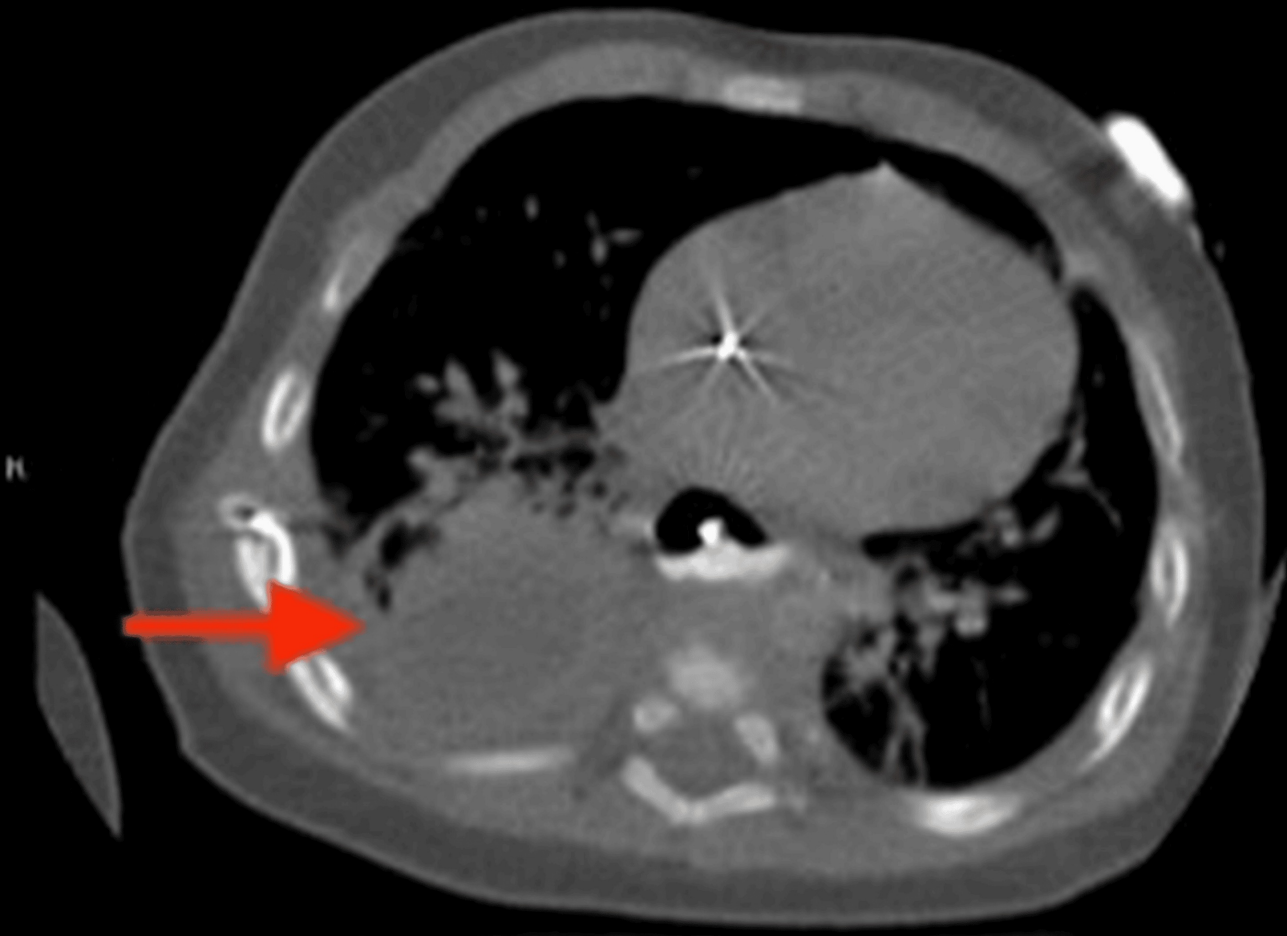
Large abscess seen on computed tomography, drained by interventional radiology.

On day two, rigid bronchoscopy and an esophagram revealed a long-gap EA (over five vertebral bodies) without visible TEF. A laparoscopic gastrostomy tube was placed. By two months, the gap had decreased to 2.5 vertebral bodies (Figure 2), leading to EA repair at three months, which included Collis gastroplasty, Nissen fundoplication, and primary esophageal anastomosis. The transanastomotic tube and chest tube were left to drain the mediastinum. On postoperative day 23, gastric output was observed through the chest tube, and a large abscess was identified on CT (Figure 3), leading to the diagnosis of an esophageal leak.

**Figure 4:**
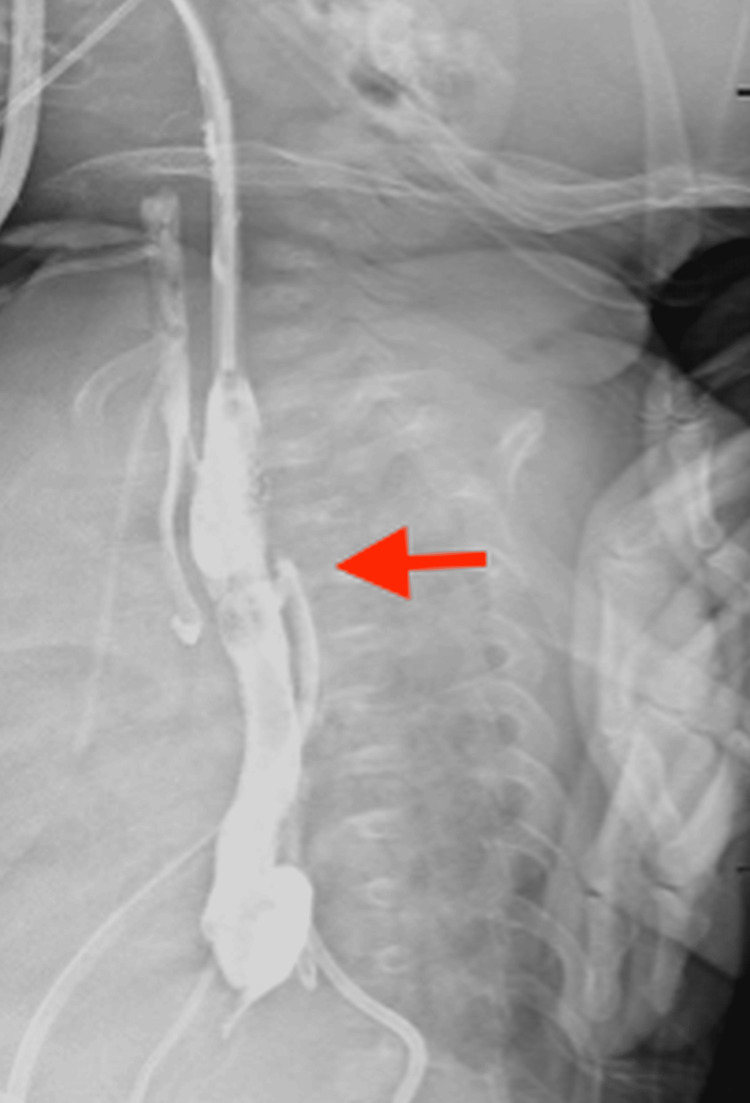
Contrast study demonstrating a proximal tracheoesophageal fistula.

**Figure 5:**
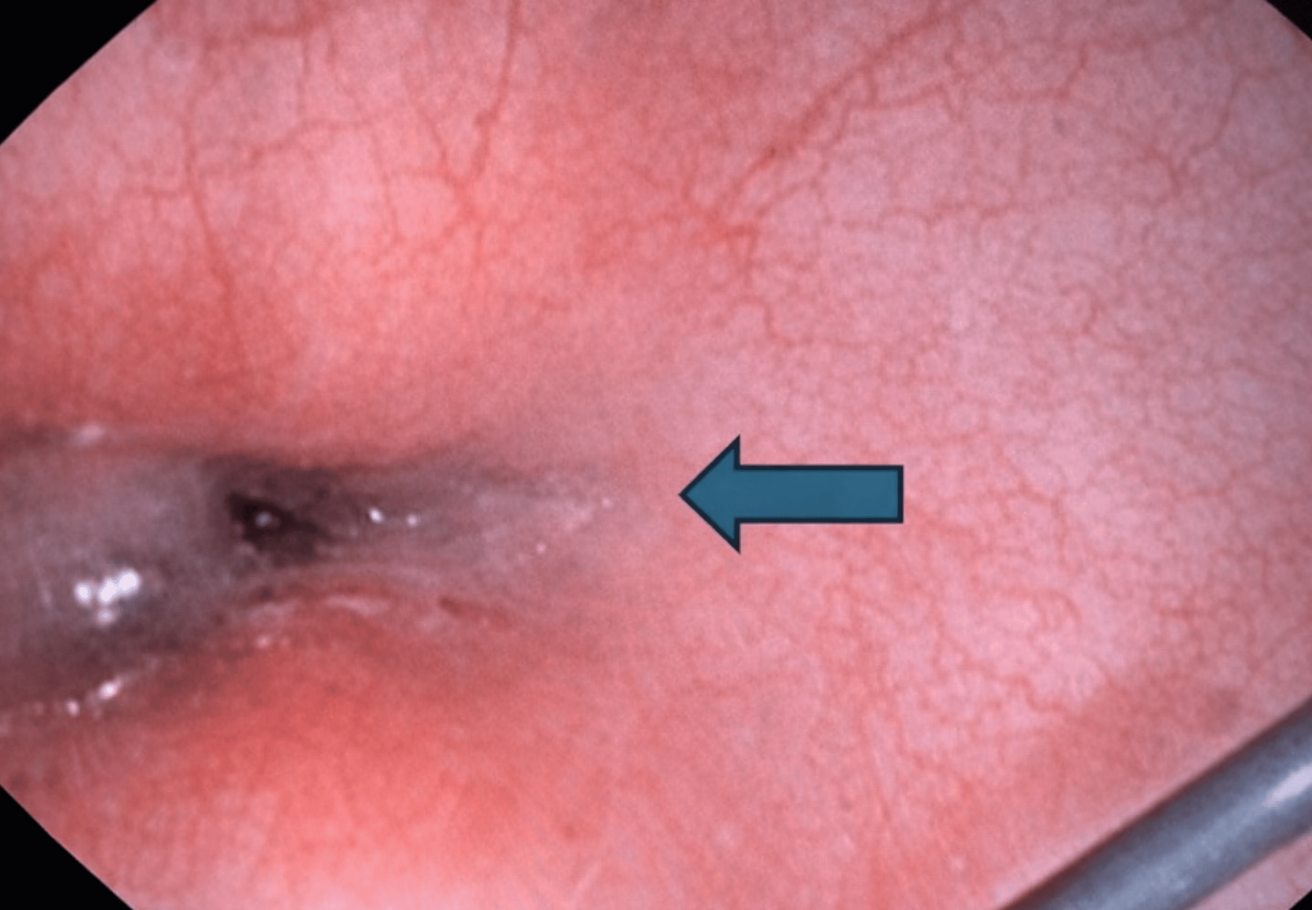
Fistula identified during bronchoscopy, 2 cm proximal to anastomosis.

At four months, a barium esophagogram revealed a small amount of contrast extravasation at the anastomotic site, with opacification of the trachea and bronchi and contrast draining into the right chest tube, indicating a previously undetected TE fistula (Figure 4). Despite this, no fistula was found on the third bronchoscopy. A fourth bronchoscopy at five months identified a small proximal TEF, confirming a diagnosis of type B EA (Figure 5). The fistula was successfully repaired through a cervical approach, and follow-up imaging confirmed resolution with no further evidence of TEF.

## Discussion

EA with a tracheoesophageal fistula is a complex congenital malformation with various types based on the gross classification. Type B EA, although rare, can present with subtle clinical features that may be missed during initial evaluations [5]. In this case, the patient's initial diagnosis of type A EA was supported by the coiled NG tube at the level of T4, which is a common sign of EA. However, the discovery of a type B TEF, detected later on imaging, highlights the challenges in diagnosing these anomalies [7]. The failure to identify the TEF early could have delayed appropriate management, leading to significant morbidity [8]. This case highlights the importance of maintaining a high index of suspicion and the necessity of repeated imaging and thorough bronchoscopic evaluation to ensure an accurate diagnosis and timely intervention.

## Conclusions

Type B esophageal atresias, though rare, can present insidiously and may be missed on initial diagnostic workups. This case emphasizes the importance of maintaining a high index of suspicion and highlights the role of repeated imaging and thorough bronchoscopic evaluation in accurately diagnosing complex esophageal anomalies. Early recognition and surgical intervention are critical for improving outcomes in neonates with EA and a tracheoesophageal fistula.
